# Molecular Signature of miR-34a/NEAT-1/p53 Axis in Mycosis Fungoides

**DOI:** 10.1155/2024/3163839

**Published:** 2024-08-16

**Authors:** Reham Fares, Shimaa M. Elasmer, Abeer Khalefa A., Olfat G. Shaker, Samar M. El-Tahlawi, Ahmed Sabri, Sara M. Yaseen

**Affiliations:** ^1^ Department of Medical Biochemistry and Molecular Biology Faculty of Medicine Fayoum University, Fayoum, Egypt; ^2^ Department of Clinical and Chemical Pathology Faculty of Medicine Fayoum University, Fayoum, Egypt; ^3^ Department of Physiology Faculty of Medicine Zagazig University, Zagazig, Egypt; ^4^ Department of Medical Biochemistry and Molecular Biology Faculty of Medicine Cairo University, Cairo, Egypt; ^5^ Department of Dermatology Faculty of Medicine Cairo University, Cairo, Egypt; ^6^ Fayoum General Hospital, Fayoum, Egypt; ^7^ Department of Dermatology, STDs & Andrology Faculty of Medicine Fayoum University, Fayoum, Egypt

## Abstract

**Background:**

Mycosis fungoides (MF) is a type of cutaneous T-cell lymphoma where red rash exists on the skin. Understanding the role of miRNAs and ncRNAs in p53-response has become an open discussion, as they can regulate p53 or its downstream targets, and ncRNAs themselves.

**Objectives:**

To evaluate the serum levels of NEAT-1, miR-34a, and p53 in MF patients and its relation to healthy controls to indicate whether it has a potential role in the pathogenesis of the disease. *Subjects and Methods*. This prospective case-control study was carried out on 75 subjects subdivided into two groups, 35 MF patients (stages 1 and II) and 40 matched healthy controls. Their clinical investigations and serum biomarkers (NEAT-1, miR-34a, and p53) were measured.

**Results:**

There were significant elevations in the expression levels of both NEAT-1 (5.10 ± 1.16) and p53 (277.28 ± 62.02) in the serum of MF patients in comparison with controls (1.01 ± 0.031) and (194.29 ± 16.039), respectively, while the level of miR-34a tends to decrease in MF patients (0.24 ± 0.15). There are no significant difference between MF stages and the level of miR-34a, while in NEAT-1 and p53, there are significant differences with *p* value <0.05 between the stages and the biomarkers. There is a positive correlation between the %BSA and miR-34a and a slightly positive correlation between NEAT-1 and P53 with (*r* = 0.353, *p*=0.037) and (*r* = 0112, *p*=0.05), respectively. There were also negative correlations between disease duration and NEAT-1 with (*r* = −0.341, *p*=0.045) and between B2 microglobulin level and p53 (*r* = −0.373, *p*=0.027).

**Conclusion:**

The combination of miR-34a, NEAT-1, and p53 may be considered as potential biomarkers that play an active role in the disease process of MF for helping in its early diagnosis and stage identification as well.

## 1. Introduction

Mycosis fungoides (MF) is considered as one of the rare incurable types of cutaneous T-cell lymphoma (CTCL). It is characterized mainly by gradual progression and infiltration of the skin with patches, plaques, or nodules to tumors and erythroderma. The complexity of MF may be diagnosed as a cancer of T-cells [1, 2].

Noncoding RNAs (ncRNAs) show a vital role in the pathogenesis, diagnosis, and treatment options for various diseases. It has been demonstrated that ncRNAs, including microRNAs (miRNAs) and long noncoding RNAs (lncRNAs), have functional roles in both the physiological and pathological processes by regulating the expression of their target genes [3–5].

Studies showed the vital role of miR-34a and lncRNA NEAT-1 in various inflammatory and skin disorders in general such as psoriasis, vitiligo, and others [6–8]. Also, the overexpression of NEAT-1 has been found to be linked to the process which promotes tumor growth, invasion, and metastasis [9].

In addition, it has been reported that the p53 gene, also known as TP53, has a potential role in regulating cell growth and preventing the formation of cancerous cells. It may also aid in suppressing the formation and progression of tumors [10–12].

The exact role of microRNAs and lncRNAs in MF remains unclear. Thus, it seemed convenient to investigate the expression levels of miR-34a and NEAT-1 in MF to figure out its possible role in its pathogenesis comparing MF patients with healthy controls. In addition, we investigated the role of p53 gene as a tumor suppressor gene.

## 2. Patients and Methods

This is a prospective case-control study where seventy-five subjects were enrolled and subdivided into two groups.

- Group 1 includes 40 healthy controls
- Group 2 includes 35 MF patients

The study was conducted at the outpatient clinic, Dermatology Department, Faculty of Medicine, Fayoum University, where ethical approval was obtained from the Ethical Committee of the Faculty of Medicine, Fayoum University, on 14/06/2023 with number M-660. A written consent from each subject before the beginning of the study was also obtained.

For selecting patients diagnosed with MF, one lesional skin 4 mm punch biopsy was taken. The specimens taken were processed and stained with *H* and *E* for light microscopic examination as well as immunohistochemical steps.

All patients had dermatological assessment for identifying the stage/extent of MF. Ultrasound investigations were performed among all individuals to check lymph nodes' existence. Skin types and clinical scoring were defined by using the Original Mycosis Fungoides Cooperative Group staging system for cutaneous T-cell lymphoma (CTCL) [13].

Some people inherit DNA mutations that increase their risk for some types of cancer from a parent. Having a family history of lymphoma does seem to increase the risk of lymphoma. Family history means that one or more family members (1st or 2nd generation) have MF.

### 2.1. Inclusion Criteria

Adult patients aged between 20 and 69 years with MF were included in the study.

### 2.2. Exclusion Criteria

Patients with any other or autoimmune/inflammatory skin diseases were excluded from the study. Patients with history and disease activity of vitiligo were not included. Pregnant and/or lactating females were also excluded. MF patients with late tumor stage or with history of solid or hematological malignancy as leukemia were also excluded from the study. As for treatment history, a patient who received treatment of MF for the past one month was excluded.

### 2.3. Molecular Biology Techniques for NEAT-1 and miR-34a

For the expression of NEAT-1 and miR-34a, certain procedures were followed:

- RNA was extracted from serum using Qiagen, Valencia, CA, USA
- RNA samples were subjected to RNA quantitation and purity assessment using the NanoDrop® (ND)-1000 spectrophotometer (NanoDrop Technologies, Inc. Wilmington, USA)
- The process of the reverse transcription (RT) which was carried out on total RNA in a final volume of 20 uL RT reactions using the miScript II RT kit (Qiagen, Valencia, CA, USA)
- Reaction mix was prepared according to the QuantiFast SYBR Green I PCR Kit for delivering fast and specific quantification of the cDNA target by real-time PCR using SYBR Green I detection
- Quantitative real-time PCR (qPCR) for detection of mature miRNA took place
- Results were calculated

Melting curve analyses were performed to validate the specific generation of the expected PCR product. As there is no known control miRNA in serum, SNORD 68 was used as an endogenous control. Also, for long noncoding NEAT-1, GAPDH was used as an endogenous control. The expression levels of miR-34a and NEAT-1 were calculated relative to the control samples (used as the calibrator sample) using the formula (2^−ΔΔCT^) and were expressed as a fold change.

### 2.4. Laboratory Assessment and Quantitation of p53 in Serum

For evaluating the level of p53 in serum, 5 ml blood samples were collected from patients and controls and were to be assessed by enzyme-linked immunosorbent assay (ELISA). Serum was then separated by centrifugation and stored at −80°C. The ELISA kit was supplied by SunRed Biotechnology Company, Catalog No: 201-12-1542.

### 2.5. Statistical Analysis

Data were organized and analysed using Statistical Package of Social Science (SPSS 17.0) on Windows 8.1. Data were subjected to the Kolmogorov–Smirnov test to determine the distribution and method of analysis. Categorical variables are shown as percentages, and the Chi-square test was used to compare demographic data (gender). For normal quantitative parametric data, Student's *t*-test is used to compare the measurement of 2 independent groups and the one-way ANOVA test was used for comparing more than 2 independent groups.

While for nonparametric data, the Kolmogorov–Smirnov test was used, and the Mann–Whitney *U* test was used to compare outcomes between two independent groups. For measuring the correlation between qualitative data, the bivariate Pearson correlation test was used to find out the association between different groups with a two-tailed test to test the significance. Sensitivity and specificity tests were generated for testing a new test with the ROC (receiver operating character) curve. *p* value <0.05 was considered as a cutoff value for significance.

## 3. Results

### 3.1. Demographic and Clinical Analysis

The patient's age ranged between 20 and 69 years and for the healthy controls between 20 and 60 years. There was no statistically significant difference between the two groups as regards to the age where *p* value >0.05. There was also no significant difference as regards to the gender. As for the disease duration, the range is between 0.16 (3 months) and 20 years as maximum with a median of the duration of 3 years. The skin types of all patients were III and IV.  Table 1 shows the demographic and clinical characteristics of MF patient groups and healthy controls.

Based on the CTCL classifications, the MF score was calculated, and accordingly, patients were classified into the following stages:

- IA: 4 (11.4%)
- IB: 13 (37.2%)
- IIA: 18 (51.4%)

As a laboratory investigation, the B2 microglobulin level was measured showing that the mean level was 2.20 ± 1.0 for all patients.

Ultrasound investigations were performed for all MF patients showing the existence of lymph nodes. Our findings show that 20 (57.1%) patients were normal with no lymph nodes, while 15 (42.9%) patients had enlarged lymph nodes.

### 3.2. Expression of miR-34a, NEAT-1, and p53 in Serum

**Figure 1:**
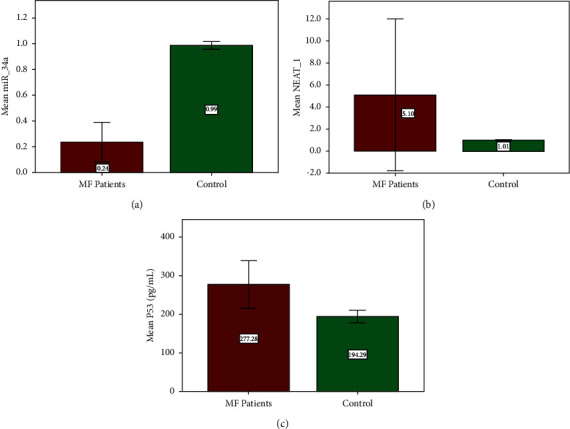
Comparison of concentration levels of serum biomarkers between MF patients' group and healthy controls: (a) miR-34a; (b) NEAT-1; (c) p53.

We then measure the levels of miR-34a, NEAT-1, and p53 in serum among both groups. Results revealed that there was a highly statistically significant difference in the patients' group when compared to the control group regarding the levels of NEAT-1 and p53 with mean and standard deviation of 5.10 ± 1.16 and 277.28 ± 62.02 for the MF patients' group and 1.01 ± 0.031 and 194.29 ± 16.039 for healthy controls with *p* values 0.001 and 0.0001, respectively. While the expression level of miR-34a tends to decrease in MF patients 0.24 ± 0.15 than that in healthy individuals 0.99 ± 0.029 with a significance level of *p*=0.0001 (Figure 1).

Table 2 shows the relationship between miR-34a, NEAT-1, p53, and clinical/descriptive data among MF patients' groups.

**Figure 2:**
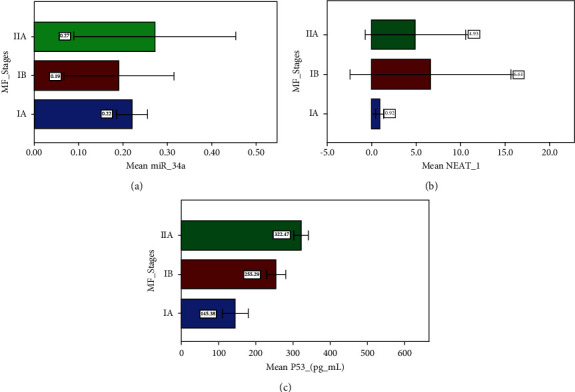
Relationship between the stages of MF and the serum biomarkers among the studied groups: (a) the expression level of miR-34a; (b) the expression level of NEAT-1; (c) the concentration level of p53.

After evaluating the expression levels of the biomarkers in serum, results revealed that there were no significant difference between miR-34a and the stages of MF where *p* value >0.05 (Figure 2(a)).

While in the expression level of NEAT-1 and the stages of MF, results revealed that the mean and standard deviation of stages are 0.92 ± 0.45, 6.61 ± 2.50, and 4.93 ± 1.32 for IA, IB, and IIA, respectively There are statistical significance differences between stages IA and IB (*p* = 0.042), IA and IIA (0.003), and IB and IIA (0.003) (Figure 2(b)).

Figure 2(c) shows the concentration level of p53 and the stages of MF where p53 tends to increase as the stage gets higher. The mean and standard deviation are 145.38 ± 35.19, 255.29 ± 25.38, and 322.47 ± 19.87 for IA, IB, and IIA stages, respectively. Statistical significance differences exist between stages IA and IB (0.004), IA and IIA (0.0001), IA and IIB (<0.0001), and IB and IIA (0.0001).

### 3.3. Correlation between Biomarkers and Different Characteristics in the MF Group

**Figure 3:**
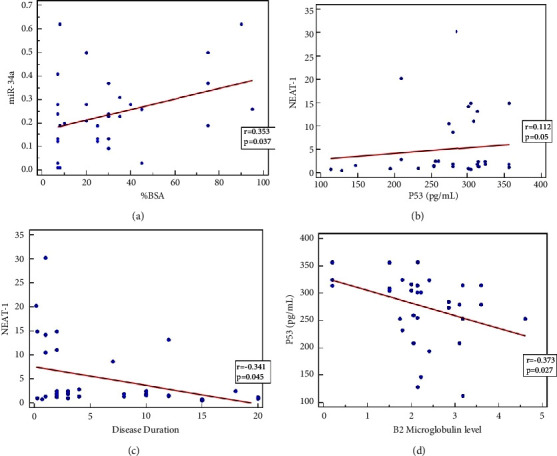
Correlation between the serum biomarkers among MF patients: (a) miR-34a and %BSA; (b) NEAT-1 and p53; (c) NEAT-1 and disease duration; (d) B2 microglobulin level and p53.

After measuring the bivariate correlation using the Pearson *R* test between miR-34a, NEAT-1, and p53 serum biomarkers and descriptive/laboratory as well as clinical data among MF patients, results showed that there is a significant positive correlation between the %BSA and miR-34a with (*r* = 0.353 and *p*=0.037) and a slightly positive correlation between NEAT-1 and p53 (*r* = 0.112 and *p*=0.05) (Figures 3(a) and 3(b)).

Also, results revealed that there are negative correlations between the disease duration and NEAT-1 with (*r* = −0.341 and *p*=0.045) and between B2 microglobulin level and p53 (*r* = −0.373 and *p*=0.027) (Figures 3(c) and 3(d)).

There is no correlation between p53 and miR-34a where *p* value = 0.441. No other correlations found between miR-34a and (age; disease duration; B2 Microglobulin level) where *p* value >0.05. Also, there are no correlation between NEAT-1 and age and B2 microglobulin level and the extent of the disease. For the correlation between the concentration level of p53 and other descriptive/laboratory and clinical data, results show that no correlation exists where *p* value >0.05.

## 4. Discussion

Mycosis fungoides is a type of cutaneous T-cell lymphoma where red rashes appear on the skin [1, 14].

In the present study, we evaluated the expression levels of miR-34a, NEAT-1, and p53 among MF patients comparing the results with healthy controls and showing their relations with clinical data. Findings revealed that the expression level of miR-34a tended to decrease in MF patients when compared to healthy controls, while NEAT-1 and p53 levels were higher in MF patients than that in controls reporting a significant difference regarding the biomarkers between both groups.

Agreeing to our findings, based on the comparative study of different miRNAs on frozen samples of 14 early MF patients and 15 inflammatory dermatitis cases using miRNA microarrays, authors showed that the level of miR-34a tended to downregulate in MF samples when compared to controls [15].

Back in 2014, a study examined the importance of miRNAs in early MF and comparing it to atopic dermatitis and advanced cutaneous T-cell lymphoma where authors showed that the expression level of miR-34 tended to decrease among 13 patients with MF which could be a vital biomarker for the progression of the disease [16].

However, some studies contradicted to our results, showing that miR-34a was upregulated and concluding that miR-34a could be a potential and promising biomarker in the molecular pathogenesis of tumor MF and as a candidate oncogenic molecule [17–20].

NEAT-1 promotes lymphomagenesis and B-cell proliferation through a MYC-regulated mechanism in diffused large B-cell lymphoma, and it has been reported to be overexpressed in patients with diffused large B-cell lymphoma [21, 22].

In 1994, authors showed that 14 patients with early stages of MF (premycotic erythema and second stage plaques) showed overexpression of p53 protein in MF patients. Also, p53 levels were highly overexpressed in 21 patients with the advanced stage of MF (third stage plaques and tumors) with a significant difference when comparing to healthy controls [23].

Also agreeing to our findings, on 68 MF/SS patients, it was reported that the level of p53 was high in general but higher in late stages and tumor MF lesions than in early stages and lesions [24].

To the best of our knowledge, this is the first study that investigates the combination of the serum levels of miR-34a, NEAT-1, and p53 in MF patients based on MF stages.

Increasing evidence has shown that lncRNAs act as a competing endogenous RNA by sponging microRNAs to alter the expression levels of their target genes in the development of cancers. It has been reported that the elevation of NEAT1 in cancer cells promotes cell growth, migration, and invasion and inhibits cell apoptosis [25].

NEAT-1 expression is upregulated in many human malignancies, including oesophageal, gastric, and lung cancers. In CSCC tissues, NEAT-1 lncRNA was expressed at high levels compared to healthy individuals and correlated with lymph node metastasis and TNM stage [26, 27].

A study demonstrated the role of NEAT-1 with a combination of different miRNAs in cancer diseases stating that the expression of NEAT-1 was significantly high in cancer cases and positively correlates with malignant features in general including late stages of non-small-cell lung cancer (NSCLC) [28, 29].

Studies showed that the expression level of miR-34a tended to decrease with an increasing clinical grade in gliomas [30]; development of metastases in colon cancer [31, 32]. Generally, the association of miR-34a was reported to be increasing associated with less aggressive cancer biology [33].

When analysing the concentration level of p53 across the different stages of MF disease, results revealed that there were significant differences between p53 and the stages and the concentration level of p53 tends to increase when the stage is aggressive (late).

Agreeing to our results, it has been concluded that the level of p53 was more common to be increasing in T3/T4 lesions than T1/T2 in MF/SS (Sézary syndrome) [24], high in nephroblastoma/Wilms' tumor and much higher in anaplastic cases [34], and then in breast tumor/benign carcinoma [35].

## 5. Conclusion

NEAT-1 and miR-34a with the combination of p53 could be used as diagnostic biomarkers for MF disease.

## Figures and Tables

**Table 1:** Demographic and clinical characteristics for studied groups.

Parameters	MF (*N* = 35)	Control (*N* = 40)
Age (years)	44.51 ± 14.94	38.45 ± 11.41

*Gender*		
Female	19 (54.3%)	26 (65%)
Male	16 (45.7%)	14 (35%)

*Skin_Phototype*		
III	15 (42.8%)	18 (45%)
IV	20 (57.2%)	22 (55%)

*Family_History*		
Yes	6 (17.2%)	—
No	29 (82.8%)	40 (100%)

*Associated_Diseases*		
Absent	33 (94.3%)	40 (100%)
Present	2 (5.7%)	—

*MF_Stages*		
IA	4 (11.4%)	—
IB	13 (37.2%)	—
IIA	18 (51.4%)	—

*Lymph node*		
Normal	20 (57.2%)	40 (100%)
Enlarged	15 (42.8%)	—

*Recurrence*		
Yes	4 (11.4%)	—
No	31 (88.6%)	—
Disease duration (years), median (range)	3 (0.16–20)	—
Extent of the disease (%), median (range)	25 (7–95)	—

**Table 2:** Relationship between the expression levels of miR-34a, NEAT-1, and p53 with clinical/descriptive data among MF patients group.

Parameters	miR-34a	*p* value	NEAT-1	*p* value	p53	*p* value
*Skin_Phototype*						
III (*N* = 15)	0.23 ± 0.15	0.969	6.89 ± 2.28	**0.019**	282.70 ± 65.03	0.585
IV (*N* = 20)	0.24 ± 0.16		3.75 ± 1.06		273.21 ± 61.04	

*Family_History*						
Yes (*N* = 6)	0.38 ± 0.15	0.055	10.82 ± 4.56	**0.010**	298.75 ± 49.61	0.438
No (*N* = 29)	0.21 ± 0.13		3.91 ± 0.96		272.83 ± 64.13	

*Associated_Diseases*						
Absent (*N* = 33)	0.23 ± 0.15	0.288	5.12 ± 1.22	0.696	281.75 ± 58.05	0.083
Present (*N* = 2)	0.23 ± 0.04		4.61 ± 4.09		203.50 ± 106.77	

*Recurrence*						
Yes (*N* = 4)	0.2 ± 0.17	0.609	11.92 ± 6.81	**0.030**	285.93 ± 10.50	**0.030**
No (*N* = 31)	0.24 ± 0.15		4.21 ± 0.95		276.16 ± 65.86	

*Lymph node*						
Normal (*N* = 20)	0.20 ± 0.143	0.593	6.21 ± 1.82	**0.040**	267.45 ± 65.30	**0.05**
Enlarged (*N* = 15)	0.28 ± 0.16		3.61 ± 1.17		290.38 ± 56.85	

Data are shown as mean ± SD. Bold values are significant (*p* ≤ 0.05).

## Data Availability

The data that support the findings of this study are available on request from the corresponding author. The data are not publicly available due to privacy or ethical restrictions.
